# Unraveling the complexity of ulcerative colitis: insights into cytokine dysregulation and targeted therapies

**DOI:** 10.17179/excli2025-8374

**Published:** 2025-06-06

**Authors:** Yuta Shimomori, Yoshihiro Yokoyama, Hiroki Kurumi, Kotaro Akita, Tomoe Kazama, Yuki Hayashi, Kazuhiro Mizukami, Hiroshi Nakase

**Affiliations:** 1 Department of Gastroenterology and Hepatology, Sapporo Medical University School of Medicine, S-1, W-16, Chuo-ku, Sapporo 060-8543, Hokkaido, Japan; Tel: +81-11-611-2111; 2 Department of Gastroenterology, Faculty of Medicine, Oita University,1-1 Idaigaoka, Hasama, Yufu 879-5593, Oita, Japan; Tel: +81-97-586-4411

**Keywords:** ulcerative colitis, cytokines, targeted therapy, innate immune cells, adapted immune cells

## Abstract

Ulcerative colitis (UC) is a chronic or recurrent inflammatory disease of the large intestine. Although the causes of UC are insufficiently understood, a complex interaction of several factors, including genetic factors, environmental factors, and gut microbiota, influences the onset of UC. The pathophysiology of UC involves intestinal barrier dysfunction, abnormal immune responses, and dysregulation of cytokines. Cytokine-targeted therapies have been approved for the treatment of UC, with several targeted therapies being currently available. The induction response rates range from 47.8 % to 73 %, and we often experience difficult-to-treat cases. In this review, we outlined the abnormal immune response and cytokine regulation underlying the complex pathology of UC. Moreover, we summarized the mode of action and the effects at the cellular and genetic levels of targeted therapies. A deeper understanding of the pathophysiology of UC and the effects of treatment is essential for advancing personalized medicine, which remains a key, challenging goal in the future management of UC.

## 1. Introduction

Ulcerative colitis (UC) is a chronic or relapsing inflammatory disease that affects the colon (Nakase et al., 2021[84]). Although the causes of UC are insufficiently understood, multiple factors, such as genetics, environmental factors, and gut microbiota, are intricately involved in its development and progression. These factors lead to intestinal inflammation through intestinal barrier dysfunction, abnormal immune responses, and cytokine dysregulation (Neurath, 2019[85]). The relationship between UC pathology and cytokines has been widely reported; among them, anti-tumour necrosis factor (TNF)-α, interleukin (IL)-12, IL-23, and Janus kinase (JAK) have been used as therapeutic targets. 

Medicines that target specific proteins, such as cytokines, are known as molecular-targeted drugs. These drugs are used to treat moderate-to-severe UC (Nakase et al., 2021[84]). Several molecular targeted drugs have been approved for the treatment of UC. These include anti-TNF-α antibodies (infliximab, adalimumab, and golimumab) (Reinisch et al., 2011[95]; Rutgeerts et al., 2005[99]; Sandborn et al., 2014[105]), anti-IL-12/23 p40 antibody (ustekinumab) (Sands et al., 2019[110]), anti-IL-23 p19 antibodies (mirikizumab, risankizumab, and guselkumab) (D'Haens et al., 2023[29]; Louis et al., 2024[68]; Rubin et al., 2024[97]), JAK inhibitors (tofacitinib, filgotinib, and upadacitinib) (Danese et al., 2022[30]; Feagan et al., 2021[36]; Sandborn et al., 2017[107]), anti-integrin antibodies (vedolizumab and carotegrast methyl) (Feagan et al., 2013[38]; Yoshimura et al., 2015[134]), and sphingosine-1-phosphate (S1P) modulators (ozanimod and etrasimod) (Sandborn et al., 2021[104], 2023[109]). Despite the development of various molecularly targeted drugs, their induction response rates range from 47.8 % to 73 %, and we often encounter cases that are difficult to treat (Danese et al., 2022[30]; D'Haens et al., 2023[29]; Louis et al., 2024[68]; Feagan et al., 2013[38], 2021[36]; Reinisch et al., 2011[95]; Rubin et al., 2024[97]; Rutgeerts et al., 2005[99]; Sandborn et al., 2014[105], 2017[107], 2021[104], 2023[109]; Sands et al., 2019[110]; Yoshimura et al., 2015[134]).

Differences in cytokine profiles among individuals and at each stage of the disease are thought to be factors that make treatment difficult (Nakase et al., 2022[83]).

In this review, we outline the abnormal immune response and cytokine regulation that underlie the complex pathogenesis of UC and summarise the current state of treatments targeting these factors.

## 2. Dysregulation of Immune Cells and Cytokines in Ulcerative Colitis

### 2.1. Innate immune cells and cytokines in ulcerative colitis

**Figure 1:**
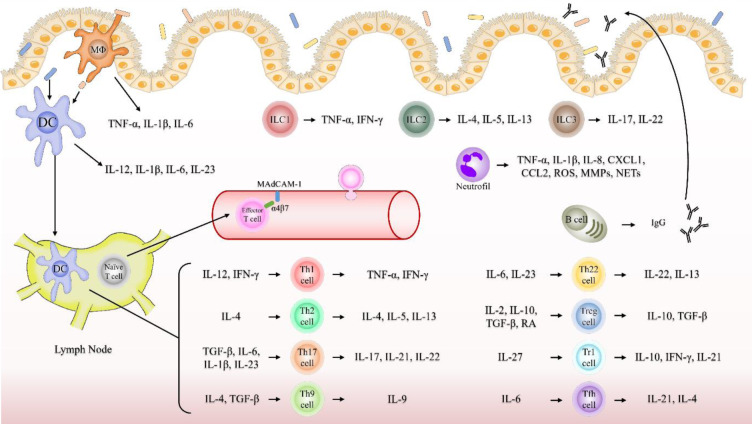
Fig. 1. Dysregulation of immune cells and cytokines in ulcerative colitis. In UC, the breakdown of the epithelial barrier promotes the influx of intestinal antigens. Consequently, pathogen- and damage-associated molecular pattern molecule levels increase, and innate immune cells promote inflammation. Inflammatory macrophages secrete pro-inflammatory cytokines, such as TNF-α, IL-1β, and IL-6, which activate immune cells and increase epithelial permeability. DCs promote the differentiation of effector T cells by producing IL-12, IL-1β, IL-6, and IL-23. Neutrophils produce ROS, MMPs, and NETs, which cause epithelial damage, and secrete chemokines to induce immune cell migration. ILCs promote inflammation by producing TNF-α, IFN-γ, IL-13, and IL-17. Naïve T cells are activated by innate immune cells and recruited to the sites of inflammation. This recruitment uses homing receptors, such as α4β7 integrin and adhesion molecules on vascular endothelial cells, such as MAdCAM-1. Activated naïve T cells differentiate into multiple types and secrete various cytokines that promote or reduce inflammation in UC. In the intestinal mucosa of patients with UC, B cells show increased IgG secretion, which promotes intestinal inflammation. Abbreviations: CCL, C-C motif chemokine ligand; CXCL, C-X-C motif chemokine ligand; DC, dendritic cell; IFN, interferon; Ig, immunoglobulin; IL, interleukin; ILC, innate lymphoid cell; MAdCAM-1, mucosal addressin cell adhesion molecule 1; MMPs, matrix metalloproteinases; MΦ, macrophage; NETs, neutrophil extracellular traps; RA, retinoic acid; ROS, reactive oxygen species; Tfh, T follicular helper; TGF, transforming growth factor; Th, T helper; TNF, tumour necrosis factor; Tr1, type 1 regulatory T; Treg, regulatory T; UC, ulcerative colitis

Innate immune cells with pattern recognition receptors (PRRs) recognise pathogen-associated molecular pattern molecules (PAMPs) and damage-associated molecular pattern molecules (DAMPs) and induce an immune response involving phagocytosis, antigen presentation, and cytokine production. In UC, the breakdown of the epithelial barrier promotes the influx of intestinal antigens. Consequently, the increased PAMP and DAMP levels in intestinal tissues stimulate innate immune cells, leading to sustained inflammation (Boyapati et al., 2016[17]; Khor et al., 2011[57]). This section explains the roles of various cells involved in the innate immunity in UC (Figure 1(Fig. 1)).

#### 2.1.1. Macrophages

CX3C motif chemokine receptor 1 resident macrophages secrete IL-10 in the normal colon (Bain et al., 2013[7]). IL-10 induces immune tolerance by promoting the differentiation and activation of regulatory T (Treg) cells and suppressing the production of inflammatory T helper (Th) cells. In patients with UC, CD14^+^ macrophages, which are highly reactive to lipopolysaccharide, andC-C motif chemokine receptor 2^+^ monocyte-derived macrophages secrete inflammatory cytokines, such as TNF-α, IL-1β, and IL-6 (Platt et al., 2010[92]; Rugtveit et al., 1997[98]). TNF-α promotes apoptosis and inflammation in epithelial cells and causes inflammation by activating immune cells and inducing apoptosis resistance in activated cells (Wajant et al., 2003[124]; Alfen et al., 2018[2]). IL-1β induces neutrophil recruitment; activation of innate lymphoid cells (ILCs), macrophages, and Th17 cells; and increased permeability of the intestinal barrier (Aschenbrenner et al., 2021[6]; Kaminsky et al., 2021[54]). IL-6 shifts the Th1/Th2 balance towards Th2 and promotes the differentiation of Th17 cells and T follicular helper (Tfh) cells (Bettelli et al., 2007[13]; Crotty, 2014[28]; Diehl and Rincón, 2002[35]). In addition, IL-6 induces the maturation of B cells and their differentiation into plasma cells (Wols et al., 2002[129]).

#### 2.1.2. Dendritic cells

Dendritic cells (DCs) take up antigens from the intestinal tract through M cells or directly, migrate to peripheral lymphoid tissues, present antigens, and induce adaptive immune responses (Yang et al., 2021[132]). DCs can be divided into plasmacytoid DC (pDC), conventional DC (cDC) 1, and cDC2 (Schraml and Reis e Sousa, 2015[111]). pDCs exert both anti- and pro-inflammatory functions. pDCs are involved in immune tolerance by inducing Treg cells (Baumgart et al., 2011[9]). On the other hand, pDCs produce inflammatory cytokines such as TNF-α, IL-6, and IL-8 in the intestinal mucosa of patients with IBD (Matta et al., 2010[73]). cDCs are involved in the differentiation of Treg cells by producing retinoic acid (RA) and activating transforming growth factor (TGF)-β and are responsible for immune tolerance in the normal intestine. Under inflammatory conditions, cDC1 produces IL-12, which is involved in the differentiation of Th1 cells. In contrast, cDC2 produces IL-1β, IL-6, and IL-23 and is involved in the differentiation of Th17 and Tfh cells (Yin et al., 2024[133]). In UC, the inflammatory effect of DCs is exacerbated by an increase in the number of antigens owing to intestinal barrier dysfunction and an increase in the expression of PRRs in DCs (Hart et al., 2005[44]).

#### 2.1.3. Neutrophils

In UC, neutrophils infiltrate the intestinal mucosa and produce reactive oxygen species (ROS), matrix metalloproteinases (MMPs), and neutrophil extracellular traps (NETs) (Biasi et al., 2013[14]; Bennike et al., 2015[11]; Kang et al., 2022[55]). ROS, including superoxide anions, hydrogen peroxides, and hypochlorous acid, are the by-products of cell metabolism (Wan et al., 2022[125]). They cause damage to cells and molecules and increase tissue destruction (Sahoo et al., 2023[103]). MMPs are a group of enzymes that degrade the extracellular matrix. In UC, they are involved in tissue degradation, the persistence of inflammatory conditions, and fibrosis (O'Shea and Smith, 2014[86]). NETs are complex networks composed of DNA, histones, and granular proteins (Long et al., 2024[66]). NETs exacerbate inflammation in UC by inducing the release of inflammatory cytokines from macrophages and cause a decline in epithelial barrier function as well as a thrombotic tendency (Long et al., 2024[66]). ROS disrupt cell membranes, and MMPs disrupt cell junctions, leading to crypt abscess formation. Neutrophils also produce inflammatory cytokines, such as TNF-α and IL-1β. Additionally, they attract both innate and adaptive immune cells by producing IL-8, C-X-C motif chemokine ligand (CXCL) 1, C-C motif chemokine ligand (CCL) 2, and calprotectin (Danne et al., 2024[31]). However, some neutrophils decrease the production of inflammatory cytokines, such as IL-6 and IL-17, and increase the production of IL-22 and TGF-β, which promote tissue healing and thus have a protective effect in inflammatory bowel disease (IBD) (Zhou et al., 2018[137]).

#### 2.1.4. Innate lymphoid cells

ILCs sense and interact with the gut microbiota to promote tissue repair and regulate the homeostasis of acquired immunity (Saez et al., 2021[102]). According to their role, ILCs are classified into ILC1s, ILC2s, and ILC3s. ILC1s depend on the transcription factor T-bet and produces TNF-α and IFN-γ in response to IL-12, IL-15, and IL-18 (Morita et al., 2016[80]). Their functions are similar to those of Th1 cells, for example responding to intracellular pathogens (Adams and Sun, 2018[1]). ILC2s depend on GATA-binding protein 3 (GATA3) to produce IL-4, IL-5, and IL-13 in response to IL-25 and IL-33 (Morita et al., 2016[80]). Their functions are similar to those of Th2 cells, including playing a role in allergic reactions, defence against parasites, and mucus production by goblet cells (Kabata et al., 2018[51]). ILC3s depend on RORγt and produces IL-22, IL-17, and TNF-α in response to IL-1β and IL-23 (Morita et al., 2016[80]). Their functions are similar to those of Th17 cells, including promoting immunity to extracellular bacteria and participating in tissue repair (Saez et al., 2021[102]). In patients with UC, ILC1 and ILC2 levels are increased (Forkel et al., 2019[39]). The increased production of IFN-γ by ILC1 and IL-13 by ILC2 may exacerbate intestinal mucosal inflammation (Bernink et al., 2013[12]; Camelo et al., 2012[20]). However, the number of natural killer (NK) p44^+^ ILC3s, which produces IL-22 and protects the epithelial barrier, decreases (Forkel et al., 2019[39]).

### 2.2. Adaptive immune cells and cytokines in ulcerative colitis

T and B cells are the main cells that play central roles in acquired immunity. Naïve T cells are activated and differentiated by antigen-presenting cells in the gut-associated lymphoid tissues (GALTs) and mesenteric lymph nodes. Differentiated T cells express homing receptors and migrate to the intestinal mucosa by interacting with adhesion molecules on vascular endothelial cells (Arseneau and Cominelli, 2015[5]). The homing receptor α4β7 integrin interacts with mucosal addressin cell-adhesion molecule (MAdCAM)-1, α4β1 integrin interacts with vascular adhesion molecule-1 and fibronectin, and αLβ2 integrin interacts with intercellular adhesion molecule (ICAM)-1 (Habtezion et al., 2016[43]). T cells that migrate to the intestinal mucosa perform their respective functions. This section describes the roles of various cells involved in adaptive immunity in UC (Figure 1(Fig. 1)).

#### 2.2.1. T helper 1 cells

Th1 cells play an important role in immune responses against many pathogens, such as bacteria and viruses, and in anti-tumour immune responses (Butcher and Zhu, 2021[19]). Naïve T cells express the transcription factor T-bet upon stimulation with IL-12 and IFN-γ, differentiating into Th1 cells (Zhu et al., 2010[140]). Th1 cells secrete IFN-γ, TNF-α, and IL-2 (Yang et al., 2007[131]). Th1 responses are hyperactivated in the intestinal mucosa and serum of patients with IBD. IFN-γ stimulates macrophages and neutrophils to induce the expression of adhesion molecules in epithelial cells, promoting the recruitment of immune cells (Li et al., 2019[64]). Th cells may acquire characteristics of other subtypes or change into other subtypes under certain conditions. They have been reported to include Th1-like Th17 cells, Th1-like Treg cells, and Th17-like Treg cells (Cohen et al., 2011[26]; Yu et al., 2021[135]). In particular, Th1-like Th17 cells are thought to be related to the pathology of UC. They have a high secretory capacity for both IL-17 and IFN-γ, which are increased in patients with UC and may contribute to mucosal inflammation (Globig et al., 2014[41]; Kamali et al., 2019[53]).

#### 2.2.2. T helper 2 cells

Th2 cells are responsible for immune responses leading to allergies and against parasitic infections (Butcher and Zhu, 2021[19]). Naïve T cells express the transcription factor GATA3 upon stimulation with IL-4 and differentiate into Th2 cells (Ho et al., 2009[47]). Th2 cells secrete IL-4, IL-5, and IL-13. In UC, Th2 cells infiltrate the intestinal mucosa, and the Th2 cytokine levels in the intestinal mucosa increase (Kałużna et al., 2022[52]). IL-13 is involved in the differentiation and proliferation of goblet and Paneth cells, supporting the production of mucus and antimicrobial peptides and the maintenance of intestinal stem cells (Steenwinckel et al., 2009[119]; Oeser et al., 2015[87]). However, Heller et al. reported that IL-13 induced apoptosis of intestinal epithelial cells, inhibited epithelial regeneration, and compromised intestinal barrier integrity in a human colon cell line (Heller et al., 2008[45]).

#### 2.2.3. T helper 17 cells

Th17 cells are responsible for the immune responses against extracellular bacteria and fungi (Zhu and Paul, 2008[139]). Naïve T cells express the transcription factor RORγt upon stimulation with TGF-β, IL-6, and IL-1β, differentiating into Th17 cells. IL-23 induces proliferation and stabilisation of Th17 cells (Bettelli et al., 2007[13]). Th17 cells secrete IL-17, IL-21, and IL-22. These cytokines induce the recruitment of neutrophils and macrophages to infected tissues and the expression of antimicrobial peptides (Chung et al., 2003[24]; Kao et al., 2004[56]). In the intestinal mucosa of patients with IBD, the expression of Th17 cells and IL-17 is higher than that in healthy individuals (Kobayashi et al., 2008[58]; Rovedatti et al., 2009[96]). IL-17 and IL-21 induce the production of MMPs by fibroblasts, causing damage to the epithelial cells (Dewayani et al., 2021[34]). However, inhibition of IL-17 is not necessarily beneficial in patients with IBD because IL-17 also plays a role in maintaining the integrity of the intestinal barrier (Lee et al., 2015[61]).

#### 2.2.4. T helper 9 cells

Th9 cells are involved in allergic reactions in the same manner as Th2 cells. Naïve T cells express the transcription factor PU.1 and IRF4 upon stimulation with IL-4 and TGF-β, differentiating into Th9 cells which secrete IL-9 (Chang et al., 2010[22]; Dardalhon et al., 2008[32]; Staudt et al., 2010[118]). Th9 cells and IL-9 levels increase in the intestinal mucosa of patients with UC (Gerlach et al., 2014[40]; Shohan et al., 2018[115]). IL-9 increases basophil and mast cell counts and promotes cytotoxic T-cell responses (Angkasekwinai and Dong, 2021[4]). 

#### 2.2.5. T helper 22 cells

Th22 cells are mainly responsible for maintaining barrier function. Naïve T cells express the transcription factor AHR upon stimulation with IL-6 and IL-23 and differentiate into Th22 cells (Rutz et al., 2013[101]; Lv et al., 2024[69]). Th22 cells secrete IL-22 and IL-13. IL-22 plays a role in maintaining the intestinal barrier function (Rutz et al., 2013[101]). IL-13 also maintains intestinal barrier function (Oeser et al., 2015[87]; Steenwinckel et al., 2009[119]). In patients with UC, the proportion of Th22 cells is decreased, which may be one of the causes of intestinal barrier dysfunction in UC (Leung et al., 2014[63]). 

#### 2.2.6. Regulatory T cells

Naïve T cells express the transcription factor Foxp3 upon stimulation with IL-2, IL-10, TGF-β, and RA, differentiating into Treg cells (Kałużna et al., 2022[52]; Yin et al., 2024[133]). Treg cells secrete IL-10 and TGF-β. These cytokines exert inhibitory effects on the inflammatory CD4^+^ T cells (Chaudhry et al., 2011[23]). In UC, Treg cells are increased in the inflamed tissues (Maul et al., 2005[74]). However, Foxp3^+^ CD4^+^ T cells, which have no inhibitory effect, and RORγt^+^ Foxp3^+^ Treg cells, which produce IL-17 and IFN-γ in addition to exerting an inhibitory effect, have been reported (Mitsialis et al., 2020[78]; Wang et al., 2007[126]). Thus, the anti-inflammatory activity of Treg cells in patients with UC is insufficient. 

#### 2.2.7. Type 1 regulatory T cells

Treg cells can be divided into the above-mentioned Foxp3^+^ Treg cells and Foxp3^-^ regulatory cells. Type 1 Treg (Tr1) cells are a subset of CD4^+^ T cells that do not express Foxp3 but secrete large amounts of IL-10 (Thomann et al., 2021[120]). Naïve T cells express the transcription factors Blimp1 and cellular musculoaponeurotic fibrosarcoma oncogene homolog (cMAF) upon stimulation with IL-27 and differentiate into Tr1 cells (Pot et al., 2011[93]). Tr1 cells secrete IL-10, IFN-γ, and IL-21. IL-10 has anti-inflammatory effects, and IL-21 acts as a growth factor in Tr1 cells (Pot et al., 2011[93]). Alfen et al. reported that IFN-secreting Tr1 cells downregulated IL-10 in patients with IBD (Alfen et al., 2018[2]). This condition is believed to be one of the causes of worsened intestinal inflammation in patients with IBD.

#### 2.2.8. T follicular helper cells

Tfh cells are found in lymphoid follicles and induce humoral immunity by promoting germinal centre (GC) responses and the differentiation of memory B cells and plasma cells (Song and Craft, 2019[117]). Naïve T cells express the transcription factor Bcl-6 upon stimulation with IL-6 produced by DCs, which then differentiate into Tfh cells (Crotty, 2014[28]). Tfh cells secrete IL-21 and IL-4. IL-21 is required for the generation of antibody-producing B cells and plays a role in maintaining B-cell proliferation in GCs (Lee et al., 2011[62]). The numbers of Tfh cells and IL-21 are increased in patients with UC (Long et al., 2020[67]). Dysregulation of the B-cell response has been implicated in UC pathogenesis (Pieper et al., 2013[91]; Wang et al., 2016[128]). Tfh cells may regulate the development of UC by regulating B-cell function (Xue et al., 2019[130]).

#### 2.2.9. B cells

B cells are divided into two types: effector B cells, which secrete IL-2, IL-4, and IFN-γ, and regulatory B cells, which secrete IL-10 (Kałużna et al., 2022[52]). B cells eventually differentiate into plasma cells and produce antibodies. Most B cells in normal intestinal mucus are immunoglobulin (Ig)A-secreting plasma cells, but the number of IgG-secreting plasma cells increases in the intestinal mucus of patients with UC (Uo et al., 2013[122]). IgA neutralises toxins and pathogens without causing inflammation because IgA secreted into the mucus cannot activate the complement cascade (MacPherson et al., 2008[70]). Uo et al. reported that in patients with UC, co-stimulation by IgG immune complexes and commensal bacteria increased the production of inflammatory cytokines, such as TNF-α and IL-1β, compared with stimulation with only commensal bacteria (Uo et al., 2013[122]). These results suggest that changes in the ratio of Igs in the intestinal mucus of patients with UC exacerbate intestinal inflammation. The increased IgG content includes autoantibodies, which are being studied for potential use as diagnostic markers and predictors of treatment response (Mitsuyama et al., 2016[79]). Recently, anti-integrin αvβ6 antibodies have been identified as autoantibodies whose level is specifically increased in the serum of patients with UC (Kuwada et al., 2021[59]). These antibodies inhibit the binding of integrin αvβ6, which is present in intestinal epithelial cells, to fibronectin in the basement membrane. However, the relationship between anti-integrin αvβ6 antibodies and the pathology of UC has not yet been clarified.

## 3. Targeted Therapies for Ulcerative Colitis

**Figure 2:**
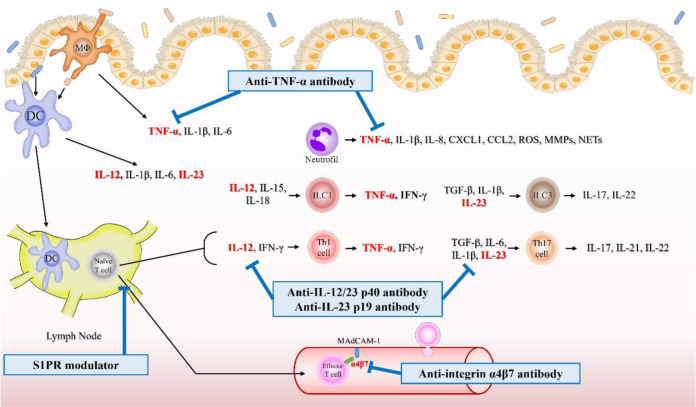
Fig. 2. Mechanism of action of targeted therapies in ulcerative colitis. Anti-TNF-α antibodies, such as infliximab, adalimumab, and golimumab, suppress inflammation by inhibiting both membrane-bound TNF-α and soluble TNF-α. Anti-interleukin IL-12/23 p40 antibodies, such as ustekinumab, bind to IL-12 p40 and IL-23 p40, preventing them from binding to their receptors and inhibiting the differentiation of Th 1 and Th17 cells. Anti-IL-23 p19 antibodies, such as mirikizumab, risankizumab, and guselkumab, inhibit the binding of IL-23 to its receptor by binding to IL-23 p19, thereby inhibiting the differentiation of Th17 cells. JAK inhibitors, such as tofacitinib, filgotinib, and upadacitinib, inhibit the activity of inflammatory cytokines by inhibiting STAT phosphorylation. The JAK inhibitors are shown in Figure 3. The anti-α4β7 integrin antibody vedolizumab and the anti-α4 integrin antibody carotegrast methyl exert anti-inflammatory effects by inhibiting the infiltration of inflammatory cells into the intestinal tract. The S1P modulators ozanimod and etrasimod exert anti-inflammatory effects by maintaining peripheral lymphocytes in the lymph nodes. Abbreviations: CCL, C-C motif chemokine ligand; CXCL, C-X-C motif chemokine ligand; DC, dendritic cell; IFN, interferon; IL, interleukin; ILC, innate lymphoid cell; MAdCAM-1, mucosal addressin cell adhesion molecule 1; MMPs, matrix metalloproteinases; MΦ, macrophage; NETs, neutrophil extracellular traps; ROS, reactive oxygen species; TGF, transforming growth factor; Th, T helper; TNF, tumour necrosis factor; S1PR, sphingosine-1-phosphate receptor

Until the 2000s, 5-aminosalicylic acid and steroids were the mainstream treatments, but since the 2010s, the era of targeted therapies has arrived. Targeted therapies have dramatically changed UC treatment. However, the approval of several drugs has created problems in selecting the best drug. When using targeted therapies, it is important to understand the targets and mechanisms of treatment. In this section, we explain these and describe the changes in gut immunity at the cellular level after drug use based on the latest evidence (Figure 2(Fig. 2), Tables 1(Tab. 1), 2(Tab. 2), 3(Tab. 3), and 4(Tab. 4); References in Tables: Boden et al., 2024[16]; Canales-Herrerias et al., 2023[21]; Hsu et al., 2023[48]; Imazu et al., 2024[49]; Massimino et al., 2022[71]; Mayer et al., 2023[75]; Mennillo et al., 2024[76]; Rath et al., 2018[94]; Smillie et al., 2019[116]; Thomas et al., 2024[121]; Wang et al., 2023[127]; Zeissig et al., 2019[136]). 

### 3.1. Anti-tumor necrosis factor-α antibodies

TNF-α exists in two forms: membrane-bound TNF-α (mTNF-α) and soluble TNF-α (sTNF-α) (Parameswaran and Patial, 2010[88]). sTNF-α binds preferentially to TNF-α receptor (TNFR) 1, whereas mTNF-α binds preferentially to TNFR2 (Wajant et al., 2003[124]). TNFR1 is ubiquitous and involved in apoptosis, cell proliferation, and secretion of inflammatory cytokines. TNFR2 is mainly expressed in immune cells and is involved in resistance to apoptosis (Wajant et al., 2003[124]). Anti-TNF-α antibodies inhibit the production of inflammatory cytokines and the proliferation of inflammatory cells by inactivating TNF-α and inducing apoptosis of activated cells (Billmeier et al., 2016[15]).

Anti-TNF-α antibodies, such as infliximab, adalimumab, and golimumab, which inhibit both mTNF-α and sTNF-α, have been effective in the treatment of UC in several controlled trials (Rutgeerts et al., 2005[99]; Sandborn et al., 2012[108], 2014[105]). In contrast, etanercept and onercept, which act mainly on sTNF-α, failed in clinical trials for Crohn's disease (CD) (Sandborn et al., 2001[106]; Van den Brande et al., 2003[123]; Rutgeerts et al., 2006[100]). Perrier et al. reported that neutralisation of mTNF-α, but not sTNF-α, was crucial for the treatment of experimental colitis (Perrier et al., 2013[89]). This highlights the importance of targeting mTNF-α in IBD treatment.

Mayer et al. reported that in patients with UC who responded to anti-TNF-α antibodies, T and B cells decreased and epithelial cells increased, but the number of innate immune cells did not change, using the spatial atlas of colon biopsy tissue using multiplexed immunofluorescence imaging technology (Mayer et al., 2023[75]). Wang et al. identified eight cell populations that were upregulated in the TNF-unresponsive group (inflammatory fibroblasts, post-capillary venules, inflammatory monocytes, macrophages, DCs, and cycling B cells) using a deep learning model based on single-cell ribonucleic acid sequence (scRNA-seq) data (Wang et al., 2023[127]). Smillie et al. identified inflammatory fibroblasts, monocytes, and cDC2s as drug-resistant cell populations (Smillie et al., 2019[116]). In their study, the three genes (*IL-13 receptor alpha 2 (IL13RA2)*, *TNF receptor superfamily member 11b (TNFRSF11B)*, and *IL11*) that showed the highest correlation with anti-TNF-α antibody resistance were specifically expressed in inflammatory fibroblasts. The IL-13Rα2 is a decoy IL-13 receptor that interferes with IL-13 function and is involved in intestinal homeostasis. Using scRNA-seq analysis, Thomas et al. reported that after adalimumab treatment, the non-responder group showed an increase in pDCs, the main producers of type I IFN, and an increase in IFN responses in epithelial and immune cells afterwards (Thomas et al., 2024[121]). Their study showed an increase in the expression of *oncostatin M receptor (OSMR)* and neutrophil chemotactic factors, such as *CXCL1* and *CXCL6*, in submucosal and lamina propria fibroblasts. In addition, upregulation of *CCL19*, a T-cell attractant, was observed in fibroblasts close to the intestinal stem cell niche. The OSMR promotes the secretion of inflammatory cytokines by fibroblasts by binding to its ligand, OSM. It is thought that the innate immune cells and fibroblasts that were increased in anti-TNF-α antibody non-responders represent the main axis of residual inflammation after anti-TNF-α antibody treatment. Furthermore, the fact that these cells increase after treatment suggests that they may also have a mechanism to escape anti-TNF-α antibodies. Therefore, it is believed that a treatment that can stop the cross-talk between innate immune cells and characteristic fibroblasts would be preferable for treating anti-TNF-α antibody non-responders.

### 3.2. Anti-IL-12/23 p40 antibody and anti-IL-23 p19 antibodies

Anti-IL-12/23 p40 antibody binds to IL-12 p40 and IL-23 p40, preventing their respective cytokines from binding to their receptors (Nakase et al., 2022[83]). IL-12 is an important cytokine that induces the differentiation of naïve T cells into Th1 cells (Zhu et al., 2010[140]). By inhibiting IL-12, Th1 cell differentiation is inhibited, and the production of Th1 cytokines, such as TNF-α and IFN-γ, is reduced. IL-23 plays an important role in maintaining and proliferating Th17 cells (Bettelli et al., 2007[13]). By inhibiting IL-23, the activation and proliferation of Th17 cells are suppressed, and the production of Th17 cytokines, such as IL-17 and IL-21, is reduced. Ustekinumab is effective and safe in patients with UC compared with a placebo (Sands et al., 2019[110]). Anti-IL-23 p19 antibody binds to IL-23 p19 and inhibits receptor binding of IL-23 to its receptor. Because it inhibits IL-23 without inhibiting IL-12, it suppresses the Th17 pathway and the production of Th17 cytokines and reduces mucosal inflammation. Mirikizumab, risankizumab, and guselkumab are more effective and safe than placebo in patients with UC (D'Haens et al., 2023[29]; Louis et al., 2024[68]; Rubin et al., 2024[97]). A head-to-head comparison of ustekinumab and risankizumab showed the non-inferiority of risankizumab in terms of the rate of clinical remission at 24 weeks and the superiority of risankizumab in the rate of endoscopic remission at 48 weeks in patients with CD (Peyrin-Biroulet et al., 2024[90]). Zhou et al. reported that the affinity and inhibitory effects of risankizumab on IL-23 were higher than those of ustekinumab (Zhou et al., 2021[138]). Meyaard et al. reported that the immune response to IL-12 increased the production of IL-10 via negative feedback (Meyaard et al., 1996[77]). These findings explain the superiority of risankizumab. In addition, because anti-IL-23 p19 antibodies do not suppress IL-12, the Th1 immune response to infections and malignancies is maintained, and these antibodies may be safer than anti-IL-12/23 p40 antibodies (Deepak and Sandborn, 2017[33]). 

Imazu et al. reported that flow cytometry of peripheral blood from patients with UC who responded to ustekinumab showed a decrease in the number of Th17 cells (Imazu et al., 2024[49]). In this study, patients with a high percentage of Th17 cells before ustekinumab treatment showed a marked improvement in clinical symptoms compared with patients with a low percentage of Th17 cells. However, in patients with CD, anti-IL-23 p19 antibodies, which inhibit the Th17 pathway more selectively, may be more effective. A head-to-head study on anti-IL-12/23 p40 and anti-IL-23 p19 antibodies is also desirable, due to the lack of existing data on UC. Wang et al. analysed gene expression in patients with CD who were resistant to anti-TNFα antibody treatment and were treated with risankizumab, based on a scRNA-seq dataset of anti-TNFα antibody treatment-resistant cases (Wang et al., 2023[127]). In this study, several modules that were upregulated in anti-TNFα antibody treatment-resistant cases were all reduced in the risankizumab response group. The innate immunity module showed the greatest reduction. In addition, analysis of the scRNA-seq data showed that the cell fractions of inflammatory fibroblasts, inflammatory monocytes, macrophages, circulating B cells, and immature intestinal epithelial cells were significantly reduced in the treatment response group. Inflammatory monocytes were the most reduced cell types. This finding supports the use of risankizumab as a next option when anti-TNF-α antibody therapy is ineffective.

### 3.3. Janus kinase inhibitors

**Figure 3:**
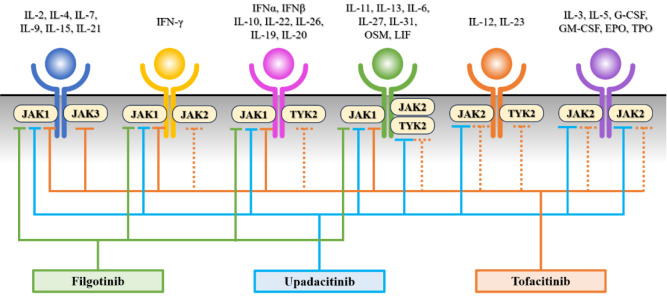
Fig. 3. JAK-STAT signalling pathway and JAK inhibitors in ulcerative colitis. The binding of cytokines to their receptors activates specific JAKs that are bound to the intracellular domain of the receptors. Consequently, the transcription factor STAT is phosphorylated, and phosphorylated STAT moves into the nucleus to regulate gene expression. Tofacitinib acts on all JAKs, filgotinib acts specifically on JAK1, and upadacitinib acts on JAK1 and partially on JAK2. Abbreviations: EPO, erythropoietin; G-CSF, granulocyte colony stimulating factor; GM-CSF, granulocyte macrophage colony stimulating factor; IFN, interferon; IL, interleukin; JAK, janus kinase; LIF, leukaemia inhibitory factor; OSM, oncostatin M; TYK, tyrosine kinase

JAKs include four types of intracellular tyrosine kinases: JAK1, JAK2, JAK3, and tyrosine kinase 2 (Clark et al., 2014[25]). When a cytokine binds to its receptor, JAKs bound to the intracellular domain of the receptor move away from each other, and their constant inhibition is released, causing them to become active. Consequently, the transcription factor STAT is phosphorylated, and phosphorylated STAT moves into the nucleus to regulate gene expression (Banerjee et al., 2017[8]). The pairing of JAK and STAT is determined by the ligand and receptor (Figure 3(Fig. 3)). JAK inhibitors exert anti-inflammatory effects by inhibiting the signalling of inflammatory cytokines and cytokines involved in the differentiation of inflammatory cells. Currently, three types of drugs are available for the treatment of UC: tofacitinib, filgotinib, and upadacitinib. Tofacitinib acts on all JAKs, filgotinib acts specifically on JAK1, and upadacitinib acts on JAK1 and partially on JAK2 (Nakase, 2023[81]). These drugs target different JAKs at different doses; therefore, there are differences in their efficacy and safety. Lasa et al. performed a systematic review and network meta-analysis to compare the relative efficacy and safety of targeted therapies in patients with moderate-to-severe UC (Lasa et al., 2022[60]). The results showed that upadacitinib was significantly more effective than all other JAK inhibitors in inducing clinical remission but also had the most adverse effects. Nakase et al. identified potential early mediators of the effect of filgotinib treatment using patient samples from a phase 2b/3 large-scale clinical trial, the SELECTION study (Nakase et al., 2024[82]). In this study, lower levels of systemic inflammatory biomarkers (C-reactive protein, serum amyloid A, and IL-6), neutrophil-associated biomarkers (calprotectin, OSM, and neutrophil gelatinase-associated lipocalin), and Th17 cytokines (IL-17A and IL-22) at week 4 were positively associated with subsequent clinical responses at week 10. In addition, filgotinib did not significantly reduce the levels of anti-inflammatory cytokines (IL-10 and TGFβ-1). Filgotinib may reduce the severity of UC by simultaneously reducing systemic inflammation, Th17 cytokines, and neutrophil inflammation while maintaining some anti-inflammatory pathways. Massimino et al. used UC-derived intestinal microvascular endothelial cell lines to analyse the differences in gene expression between tofacitinib-responding and non-responding cells (Massimino et al., 2022[71]). In this study, gene ontology analysis revealed that responding cell lines were enriched in biological processes related to JAK-STAT signalling, negative regulation of leukocyte adhesion, and epithelial barrier formation compared with non-responding cell lines. In addition, transcriptome analysis showed that tofacitinib administration reduced the population of ICAM-1 and chemokines involved in leukocyte migration in reactive cell lines. In contrast, transcripts encoding tight junction proteins were not altered by tofacitinib. This study suggests that drugs other than anti-integrin antibodies inhibit blood cell trafficking. Clarifying the effects of each drug on the vascular endothelium is thought to be one of the important factors in making treatment choices based on the pathology of UC.

Wang et al. analysed gene expression in patients with CD who were resistant to anti-TNFα antibody treatment and were treated with tofacitinib (Wang et al., 2023[127]). In this study, the analysis of scRNA-seq data showed that the cell fractions of inflammatory fibroblasts, inflammatory monocytes, macrophages, circulating B cells, and immature intestinal epithelial cells were significantly reduced in the upadacitinib response group. Inflammatory fibroblasts were the most affected cell type. Upadacitinib may be effective against populations of cells, such as inflammatory fibroblasts, that are resistant to therapy with anti-TNF-α antibodies.

### 3.4. Anti-integrin antibodies

Anti-α4β7 and anti-α4 integrin antibody preparations are used in the treatment of UC. Both exert their anti-inflammatory effects in the treatment of UC by inhibiting the migration of lymphocytes into the intestinal tract and the infiltration of inflammatory cells into the intestinal tract. Vedolizumab, an anti-α4β7 integrin antibody, and carotegrast methyl, an anti-α4 integrin antibody, are effective in patients with UC (Feagan et al., 2017[37]; Yoshimura et al., 2015[134]). Carotegrast methyl, an anti-α4 integrin antibody, has also been used in limited clinical practice because it cannot be excluded that it is a risk factor for progressive multifocal leukoencephalopathy (Yoshimura et al., 2015[134]). However, anti-α4β7 integrin antibody drugs are relatively safe, with a low risk of side effects, because they act specifically on the intestinal tract (Lasa et al., 2022[60]; Loftus et al., 2020[65]). 

Boden et al., assessed immune cell localisation after vedolizumab administration using flow cytometry (Boden et al., 2024[16]). This study showed a decrease in the numbers of naïve B cells, naïve T cells, and IgM^+^ memory B cells in the colon. Hsu et al. reported that the proportions of intestinal Th17 cells, terminal effector CD4^+^ T cells, terminal effector CD8^+^ T cells, and ILC/NK cells were reduced in vedolizumab responders using cellular indexing of transcriptomes and epitopes by sequencing (CITE-seq) analysis (Hsu et al., 2023[48]). Canales-Herrerias et al. reported that the size of GALTs and follicular tissues was reduced, and intestinal IgG^+^ plasma cells were reduced in vedolizumab responders (Canales-Herrerias et al., 2023[21]). Zeissig et al. reported that vedolizumab did not affect the number of T cells in the lamina propria, T-cell activation, or the T-cell receptor repertoire (Zeissig et al., 2019[136]). This report also demonstrated the effect of vedolizumab on innate immunity, with changes in the expression of pattern recognition receptors, chemokines, and NK molecules and a shift in macrophages from inflammatory to regulatory. Mennillo et al. reported that CITE-seq profiling of colon tissues from patients responding to vedolizumab showed a significant decrease in tissue mononuclear phagocytes, expansion of some epithelial subsets, and a trend towards a decrease in activated fibroblasts (Mennillo et al., 2024[76]). Boden et al. reported that the most significant change following vedolizumab administration was a significant decrease in cDC2 in the intestinal tract of patients who responded to vedolizumab (Boden et al., 2024[16]). Vedolizumab treatment may broadly control T cells, innate immune cells, acquired immune cells, and stromal cells. Vedolizumab is highly safe and widely controllable and may be suitable for maintenance treatment, which requires long-term stability.

In contrast, Hsu et al., reported that Th17 cells interacted with inflammatory monocytes/macrophages expressing *IL1A*, *IL1B*, *IL1RN*, *OSM*, and *CCL20* and with bone marrow DCs expressing *OSM* and were thought to be involved in amplifying and controlling inflammation in vedolizumab non-responders (Hsu et al., 2023[48]). Rath et al. reported that TNF-dependent signalling was highly activated in vedolizumab non-responders (Rath et al., 2018[94]). As interactions between Th17 cells and innate immune cells and activation of TNF-dependent signalling were observed in the vedolizumab non-responder group, treatment with IL23 p19 and anti-TNFα antibodies would be worth trying.

### 3.5. Sphingosine-1-phosphate receptor modulators

S1P is a bioactive lipid molecule that is mainly produced by erythrocytes and endothelial cells (Hla and Brinkmann, 2011[46]). There are five types of S1P receptor (S1PR), each with different signalling properties. S1PR1 is expressed in lymphocytes and mediates their migration from the lymph nodes (Matloubian et al., 2004[72]). S1PR1 is also expressed in atrial cardiomyocytes and cardiovascular endothelial cells and is involved in blood pressure and heart rate regulation (Hla and Brinkmann, 2011[46]). S1PR2 and S1PR3 are expressed in the central nervous system, vascular endothelial cells, and smooth muscle cells and are involved in vascular endothelial barrier function, neuronal migration, and vascular tension (Groves et al., 2013[42]; Shida et al., 2008[114]). S1PR4 is mainly expressed in lymphocytes and haematopoietic cells and is involved in lymphocyte migration and the regulation of DCs and Th17 cells (Brinkmann 2007[18]; Schulze et al., 2011[112]). S1PR5 is involved in the maturation of oligodendrocytes in the central nervous system and the migration of NK cells in the spleen (Comi et al., 2017[27]; Jenne et al., 2009[50]).

S1PR modulators such as ozanimod and etrasimod have recently become available for treating UC. Ozanimod is an orally administered selective S1PR1 and S1PR5 modulator that maintains peripheral lymphocytes in the lymph nodes by inducing intracellular translocation and degradation (Scott et al., 2016[113]). This inhibits lymphocyte migration into the inflamed tissue. In clinical trials, ozanimod has shown better results than placebo in both the induction and maintenance of remission (Sandborn et al., 2021[104]). Etrasimod is an oral S1PR1, S1PR4, and S1PR5 modulator that exerts anti-inflammatory effects by controlling lymphocyte trafficking. In addition, etrasimod has inhibited inflammatory cytokines, such as TNF-α, IL-1β, IL-6 and IL-17A, and increased the expression of the anti-inflammatory cytokine IL-10 in a mouse model of colitis (Al-Shamma et al., 2019[3]). In clinical trials, both the induction and maintenance of remission have been shown to be superior to those of a placebo (Sandborn et al., 2023[109]). Ozanimod and etrasimod cause bradycardia because they suppress S1PR1, which is involved in the regulation of heart rate (Becher et al., 2022[10]; Sandborn et al., 2023[109]). Because S1PR modulators are a new class of drugs, they have not yet been analysed at the molecular level, and further analysis is expected in the future.

## 4. Conclusion

This review outlined the abnormal immune response and cytokine regulation underlying the complex pathogenesis of UC. We presented the latest analyses of targeted therapies and the changes they induce at the molecular level. Treatments for UC are gradually improving, owing to high-quality clinical trials and basic studies using the latest technologies. With the wide range of drugs currently available, the greatest challenge looking to the future is to develop personalised medicine by identifying the most effective drug for each patient. Personalised medicine and deeper remission are required to further improve treatment outcomes.

## 5. Declaration

### Acknowledgments

None.

### Conflict of interest

The authors declare that they have no conflicts of interest.

### Author contributions

YS, YY, HK, KA, TK, YH, KM, and HN contributed to methodology. YS, YY, amd HN contributed to conceptualization, data curation, and formal analysis. YS and YY contributed to investigation and visualization. YS contributed to writing - original draft. HN contributed to supervision, writing - review & editing.

### Using Artificial Intelligence (AI)

No artificial intelligence (AI)-assisted technologies (e.g., Large Language Models [LLMs], chatbots, or image creations) were used in the submission process to complete the manuscript.

## Figures and Tables

**Table 1:**
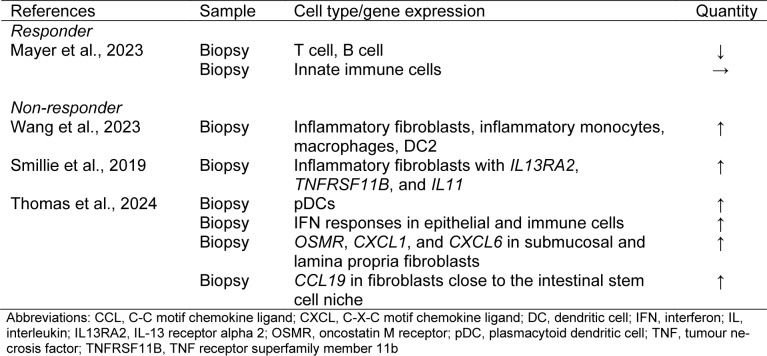
Table 1. Effects of anti-TNFα antibodies on the intestinal immunity

**Table 2:**
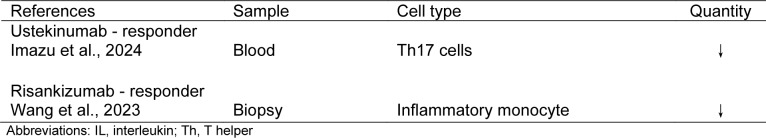
Table 2. Effects of anti-IL-12/23 p40 and anti-IL-23 p19 antibodies on the intestinal immunity

**Table 3:**
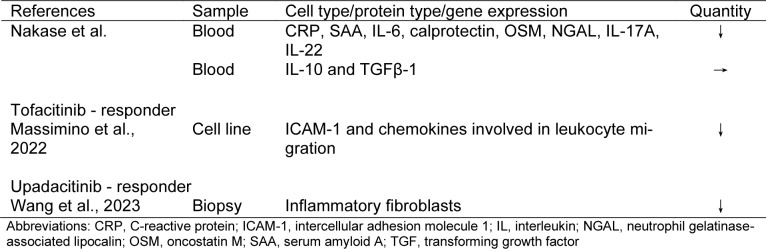
Table 3. Effects of JAK inhibitors on the intestinal immunity

**Table 4:**
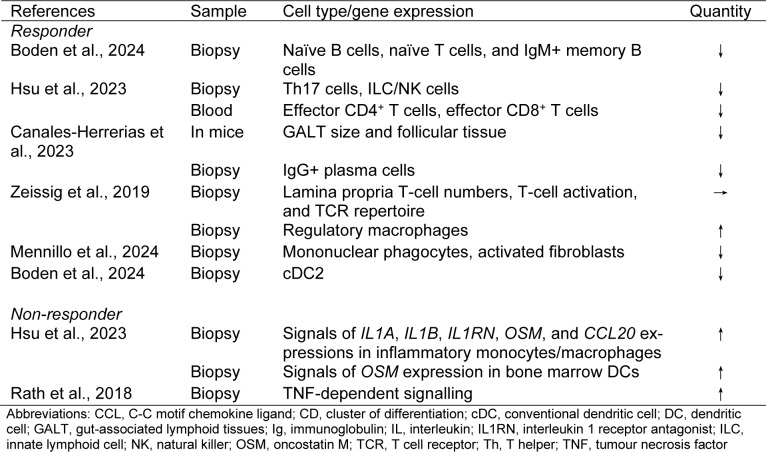
Table 4. Effects of anti-integrin antibodies on the intestinal immunity
